# The role of Dermcidin isoform-2 in the occurrence and severity of Diabetes

**DOI:** 10.1038/s41598-017-07958-3

**Published:** 2017-08-15

**Authors:** Suman Bhattacharya, Md. Mobidullah Khan, Chandradipa Ghosh, Sarbashri Bank, Smarajit Maiti

**Affiliations:** 1 Sinha Institute of Medical Science and Technology, West Bengal, India; 2 PG Department of Biochemistry, Cell and Molecular Therapeutics Laboratory, Oriental Institute of Science and Technology, Midnapore, West Bengal, India; 3 Department of Human Physiology with Community Health, Vidyasagar University, Midnapore, West Bengal, India

## Abstract

Diabetes is now epidemic worldwide. Several hundred-million peoples are presently suffering from this disease with other secondary-disorders. Stress, hypertension, sedentary life-style, carbohydrate/lipid metabolic-disorders due to genetic or environmental factors attributes to type-1 and/or type-2 diabetes. Present investigation demonstrates that stress-induced protein dermcidin isoform-2 (DCN-2) which appears in the serum of diabetic-patients play a key-role in this disease pathogenesis/severity. DCN-2 suppresses insulin production-release from liver/pancreas. It also increases the insulin-resistance. Stress-induction at the onset/progression of this disease is noticed as the high-level of lipid peroxides/low-level of free-thiols in association with increase of inflammatory-markers c-reactive protein and TNF-α. DCN-2 induced decrease in the synthesis of glucose-activated nitric oxide synthase (GANOS) and lower production of NO in liver has been shown here where NO is demonstrated to lower the expression of glucose trabsporter-4 (GLUT-4) and its translocation on liver membrane surface. This finally impairs glucose transport to organs from the extracellular fluid. Low level of glucose uptake further decreases glucose-induced insulin synthesis. The central role of DCN-2 has been demonstrated in type-1/type-2 diabetic individuals, in rodent hepatocytes and pancreatic-cell, tissue-slices, *in-vitro* and *in-vivo* experimental model. It can be concluded that stress-induced decrease in insulin synthesis/function, glucose transport is an interactive consequence of oxidative threats and inflammatory events.

## Introduction

Diabetes mellitus, a global public health problem is now emerging as an epidemic worldwide^1^. According to IDF DIABETES ATLAS (6^th^ edition), the majority of the 382 million people are suffering from diabetes. All forms of diabetes are on the surge, and the number of people with diabetes will increase by 55% by 2035^2^.

Glucose has been reported to have an important role both in the synthesis and secretion of insulin, which has been thought to be produced only in the pancreatic β-cells^3^. But, the hormone is also produced in the hepatocytes of adult mice when stimulated by glucose^4^. Thus, it self help in the maintenance of its own systemic homeostasis. Diabetes mellitus can be due to either a deficiency of insulin which is known as type-1-diabetes mellitus or an inability of insulin to control hyperglycemia, known as type-2-diabetes mellitus^5^. It has been reported that the sugar is needed to be acted upon hexokinase for the formation of glucose-6-phosphate for the insulin synthesis to follow the normal glucose homeostasis^6^.

The type I diabetes mellitus as mentioned above is however, subdivided in two categories: (i) Type-1A-diabetes mellitus (T1ADM) and (ii) Type-1B-diabetes mellitus (T1BDM)^7^. The T1ADM is reported to be caused by the destruction of the pancreatic-β cells by auto immunologic assault^8, 9^. The T1BDM on the other hand, has been reported to be induced by stress or other environmental factors. We have recently reported the appearance of a stress-induced protein of MW 11,000 kDa, identified to be dermcidin isoform-2 (DCN-2) in the circulation of the individuals with T1BDM^10^. It has been reported that the T1BDM occurred as a major form of T1DM due to dermcidin induced inhibition of glucose uptake, rather than destruction of the pancreatic β cells^11^.

Some disease conditions sharply augment the human plasma dermcidin (DCN-2). Patients suffering from acute myocardial infarction (AMI) have high level of DCN-2 in their plasma^11, 12^. And as because diabetes is the major risk factor for the genesis of AMI and atherosclerosis, so, diabetic patients (both type-1 and type-2) have high level of DCN-2 in their plasma. Stress induced genesis of diabetes in patients is reported^10^. It is also reported from our laboratory that high altitude illness may augment plasma level of DCN due to environmental stress^13^. The involvement of DCN peptide in tumorigenesis is reported^14, 15^. In all these instances stress and DCN have been correlated to the patho-physiological conditions.

In this context, It should also be mentioned here that endothelial dysfunction is an important contributor to diabetes mellitus that leads to an increase of intracellular oxidative stress due to the over production of free radicals^16–18^. Systemic stresses from exogenous and endogenous sources are of significant concern in this regard. Although stress has been reported to instigate both diabetes mellitus^19^ and hypertension^20^, the interaction between these two risk factors is poorly understood^21^ except that hypertension is found to be associated with insulin resistance and dyslipidemia^22^. As mentioned earlier that the stress induction of DCN-2, it is important to evaluate the relations between different stress-associated variables and the expression level of DCN-2.

In this regard, we report herein the level of systemic free-thiol content, TBARS and conjugated di-ene (CD) in different diabetic patients. We also report the influence of oxidative stress protein DCN-2 on the systemic production of NO and insulin in different diabetes individuals. Furthermore, it is also found that oxidative stress protein is a potent activator of the metabolic inflammatory markers C-reactive protein, malondialdehyde in these victims. It is also reported that DCN-2 can completely inhibit the sugar transport in the liver and pancreatic β-cells through the inhibition of Glut-4. The present research is absolutely important for the understanding of how systemic stress can regulate insulin level/sensitivity via the action of NO and attribute to the occurrence and severity diabetes.

## Materials and Methods

### Ethical clearance

The protocol was approved by the **Internal Review Board, Human and Animal Research Ethics Committee, Sinha Institute of Medical Science and Technology**, Kolkata, and **Department of Biochemistry, OIST, Vidyasagar University, Midnapore** on the condition that followed the approved Human Ethics Protocol strictly in accordance with 1964 Helsinki declaration and no deviation in the study was allowed without the prior written permission of the board.

Patients with diabetes mellitus (DM) and normal volunteers, who served as control, participated in the study under the strict supervision of registered endocrinologist.

**Randomly and consecutively selected total 30 (Male = 15, Female = 15) diabetic patients (screened and diagnosed by an endocrinologist) between the ages 35–60 years with characteristics hyperglycemia who were came to the Endocrinology Unit for the treatment in concerned medical colleges cum hospitals in Midnapore and Calcutta cities**. These patients have no reported complications of chronic cardiac, nephritic, peripheral vascular or chronic infectious diseases. **All the participants signed an informed consent form before their participation in the study. Only willing participants were selected for the study**. All These patients were confirmed type-1 diabetes mellitus by (I) they had plasma glucose level ≥ 200 mg/dL after overnight fasting, (II) they had plasma insulin level ≤ 10 µU/mL and (III) these patients suffering from this condition for more than 1 year. Diet controls along with physical exercises were the main strategies used by these subjects for the control of hyperglycemia. As it was essential for the intended study that these volunteers were free of liver diseases, as much as could be ascertained, volunteers with history of hepatitis, fatty liver, hepatomegaly, cirrhosis, neoplastic conditions were excluded from the study. All subjects were asked to avoid any medication including acetyl salicylic acid at least for 3 weeks before they were asked to participate in the study. All the selected patients had BMI ranging between 19 to 21.2 kg/m^2^.

The required approval was obtained for the use of animals in the study by the **Internal Review Board, Human and Animal Research Ethics Committee, Sinha Institute of Medical Science and Technology**, consisting of a special committee for animal care and their use that oversaw the welfare, care and nutritional requirements for all the animals used in the study. The committee had a permanent certified veterinarian whose duty is to ensure that the all the animals were free from any diseases as stipulated by the Animal Right Group. The animal care and all animal related experiments were strictly performed in accordance with the guidelines approved by the Ethics Review Committee in the presence of a member of the Animal Right Group and under the supervision of the veterinarian. After the termination of the study, the animals were sacrificed by euthanasia in a carbon dioxide chamber.

White albino healthy mice (20–25 gm each), Swiss strain, irrespective of gender were used for the study. These inbred animals were fed standard laboratory chow and sterilized water was given *ad libitum*. The animals were kept under 12 h cycles of light and dark at 23 °C.

### Chemicals

Goat anti-rabbit HRP conjugated secondary antibody, o-phenylenediamine dihydrogen chloride (OPD), Bovine serum albumin (BSA), thiobarbituric acid (TBA), reduced glutathione (GSH), 5–5′-dithiobis-2-nitro benzoic acid (DTNB), C-reactive protein (CRP) measurement kits, insulin and GLUT-4 gene specific primers and Revert Aid M-MulV reverse transcriptase (MBI Fermentas) were obtained from Sigma-Aldrich (St. Louis MO). Polyvinylidene difluoride (PVDF) membranes (Immunoblot PVDF) were purchased from Bio-Rad (Hercules, CA). Insulin primary antibody (H-86), Glut-4 primary antibody (H-61) and TNF-α primary antibody were obtained from Santacruz Biotechnology Inc, (Santacruz, CA, USA). ELISA Maxisorb plates were from Nunc, Rosklide, Denmark. All other chemicals were of analytical grade.

### Collection of Blood

Nominal amount of blood were collected in plastic vials by venipuncture from the participants in sodium citrate as anticoagulant^23^ [9 vol blood: 1 vol of the anticoagulant (0.013 M final concentration)] using 19 gauge siliconized needles under the supervision of an attending endocrinologist and nurses. Written consent was obtained from each of the participants.

### Preparation of cell free plasma

Blood was drawn from both the normal and diabetic individuals as described above. Cell free plasma (CFP) from the whole blood was prepared by centrifuging the blood samples at 30,000 g for 30 min at 0 °C^23^. The supernatant was collected and used as CFP.

### Preparation of dermcidin

Dermcidin used in all experiments was prepared by repeated poly acrylamide gel-electrophoresis from the cell free plasma of acute ischemic heart disease patients in the absence of sodium dodecyl sulfate followed by the elution of protein from the gel after overnight dialysis at 4 °C^11^.

### Preparation of mice liver cell homogenate

Adult mice were killed by cervical dislocation and the entire liver was immediately excised out and placed in cold Tyrod’s buffer (pH 7.4). The homogenate of the excised liver was made in the same buffer as described^24^.

### Preparation of Islets of Langerhans from the mice pancreas

The islets of Langerhans were prepared and suspended in Tyrod’s buffer (pH 7.4) as described^25^ and were used within 1 h of the preparation.

### Assay of NO in the cell free plasma (CFP) of both normal and T1DM patients

The amount of NO in the CFP of both normal and diabetic subject was assessed by using the conversion rate of oxyhemoglobin to methemoglobin through NO using a scanning spectrophotometer (Beckman spectrophotometer). The NO content was quantitated by recording the spectral changes in the reaction mixture due to the conversion of oxyhemoglobin to methemoglobin I,e a decrease in the absorbance at 575 and 630 nm as described^26^. The quantitation of NO was independently verified by chemiluminescence method^27^.

### Determination of plasma insulin levels in both T1DM and normal subjects by ELISA

An enzyme-linked immunosorbant assay was performed as described^28^, to determine the plasma level of insulin in T1DM and normal subjects. Briefly, 50 µl of plasma from both the T1DM and normal subjects were incubated with equal volume of phosphate buffer saline (PBS) in the assay plate for overnight at 4 °C. The standard ELISA protocol was fallowed. The development of color was determined at 450 nm. The amount of insulin present in the sample was determined by an ELISA reader.

### Identification of dermicidin by immunoblot and its quantitation by ELISA in the cell free plasma of both T1DM and normal subjects

The presence of dermcidin in the cell free plasma of both normal and T1DM subjects were determined by immunoblot technique^29^. The plasma samples were subjected to SDS-polyacrylamide gel electrophoresis (SDS-PAGE) sample buffer (1:1) and stained with coomassie brilliant blue^30^. The transfer of the separated protein bands in the plasma samples were next carried out electrophoretically to a PVDF membrane, and subjected to immunoblotting using anti-dermcidin antibody (1:500). The membrane was blocked with 5% BSA in TBS. After incubation with a HRP-linked goat anti-rabbit secondary antibody, dermcidin protein bands were visualized by using an enhanced chemiluminescence detection system (Thermo Scientific Rockford, IL).

In parallel experiment, the amount of dermcidin level in both the plasma samples was quantitated by ELISA by using anti-dermcidin antibody.

### Determination the role of dermcidin on blood glucose, NO and insulin level in normal adult mice

As dermcidin isoform-2 (DCN-2) is a potent inhibitor of all known forms of nitric oxide synthases^31^, and inhibition of NO synthesis results overt hyperglycemia due to impairment of insulin synthesis^32^, studies were conducted to determine the role of dermcidin on blood glucose, NO and insulin level in normal mice. Swiss white albino mice irrespective of the gender were used in the study. These animals were inbred in our animal facility. Ten mice used were divided into two equal groups.

1. Normal control group injected with only 0.9% NaCl.
2. Normal experimental group injected with DCN-2 protein, in the amounts similar to the plasma DCN-2 level in T1DM victims having blood glucose >340 mg/dL.

The blood glucose level was determined by a glucose analyzer. The plasma NO and insulin level in both of these group were quantitated by methemoglobin method as described^26^ and by ELISA^28^ by using anti-insulin antibody respectively.

### Determination the role of dermcidin on glucose activated nitric oxide synthase (GANOS) in the liver cell homogenate of adult mice

Studies were conducted to determine the role of DCN-2 on the synthesis of GANOS in the liver cell homogenate of adult mice^33^. The liver cell homogenate was prepared and suspended in Tyrod’s buffer (pH 7.4) as described in the Materials and Methods, and were incubated for 30 min at 37 °C with or without glucose and in the presence or absence of DCN-2, in the amounts similar to the plasma DCN-2 level in T1DM subject. The synthesis of GANOS was determined by *in vitro* translation of mRNA^34^ and was quantitated by ELISA^28^. In some of the experiments 0.2 µM DCN-2 was also added to the reaction mixture to inhibit the synthesis of GANOS.

In parallel experiment, the presence of GANOS in the liver cell homogenate treated with or without DCN-2 was determined by immunoblot technique^25^.

### ELISA of Insulin, and the RT-PCR of proinsulin genes I and II and GLUT4 gene

Insulin synthesized in the pancreatic islets of Langerhans in the presence of 0.02 M glucose and in the presence and absence of DCN-2 was quantitated by ELISA as described before^28^. The gene expression of proinsulin genes I and II were determined by cDNA preparation with gene specific primer as described earlier^4^. The mRNA of Glut-4 was isolated from the glucose treated hepatocytes in the presence or absence of dermcidin by Trizol method^35^. RT-PCR was done using the primer (5′-CTG GGC TGA TGT GTC TGA CG-3′) as forward and (5′-CAC ACC AGC TCC TAT GGT GG-3′) as reverse primer. Each cycle for PCR consisted of 30 second at 95 °C, 45 second at 58 °C, and 60 second at 72 °C and 35 cycles was carried out. The cDNA was synthesized from the mRNA using Revert Aid M-MulV reverse transcriptase (MBI Fermentas) as instructed by the manufacturer.

### Determination of glucose uptake

Both liver cell and β-cell of pancreas were incubated with insulin in the presence and absence of dermcidin for 2 h followed by stimulation with insulin (240 nM) for 30 min. 2-deoxy-D-^14^C glucose (0.4 nM/mL) was added to each incubation mixture 10 min before termination of the experiment. Cells were washed with ice cold Krebs buffer (pH 7.4) in the presence of 0.2 mM phloretin. Glucose uptake was measured as described^36^.

### Estimation of malondialdehye (MDA) levels in the cell free plasma of both T1DM and normal subject

As oxidative stresses and inflammatory responses are the key regulatory factors in the pathogenesis of T1DM^37, 38^ studies were conducted to determine the MDA levels, one of the oxidative stress product in the CFP of both diabetic and normal individuals. The MDA assay was conducted following the protocol as described^39^ with a slight modification. The MDA is measured and calculated using the molar extinction coefficient of MDA (1.56 × 10^5^ cm^2^/mmol)^40^.

### Estimation of C-reactive protein (CRP) in the cell free plasma of T1DM and normal subject

C-reactive protein in human serum was assessed *in vitro* by qualitative and semi-quantitative rapid Latex Slide Tests using Diagnostic Reagent kit (Sigma Aldrich) following the supplier’s protocol.

### Estimation of Non Protein Soluble Thiol (NPSH)

The NPSH in serum sample is determined by standard DTNB (5, 5′- dithiobis-2-nitrobenzoic acid) with a little modification^41^. In brief, the protein is precipitated by trichloroacetic acid and clear supernatant is added to 0.8 M Tris-EDTA (*p*H 9) buffer containing 20 mM EDTA and 5 mM DTNB. The contents were mixed well and absorbance read at 412 nm. The level of NPSH is determined against L-Cysteine hydrochloride standard curve.

### Dermcidin induced synthesis of TNF-α in hepatocytes and estimation of TNF-α in the cell free plasma of diabetic and normal volunteers

Dermcidn induced synthesis of TNF-α in liver cell homogenate of normal mice was quantified by ELISA by using monoclonal antibody according to the method described before^28^. Plasma level of TNF-α in both normal and diabetic patients were also determined by the same.

### Determination of Blood glucose level

The blood glucose level was determined by a glucometer (Behringer).

### Statistical analysis

All experiments were repeated at least three times. Data are expressed as means ± the standard error of the mean. The statistical analysis of differences between experimental groups was performed by one-way analysis of variance (ANOVA). A p value of <0.05 was considered statistically significant. Further statistical analysis was performed by Bonferroni’s multiple-comparison tests.

## Results

### Plasma level of dermcidin in type I diabetes mellitus (T1DM) subjects

**Figure 1:**
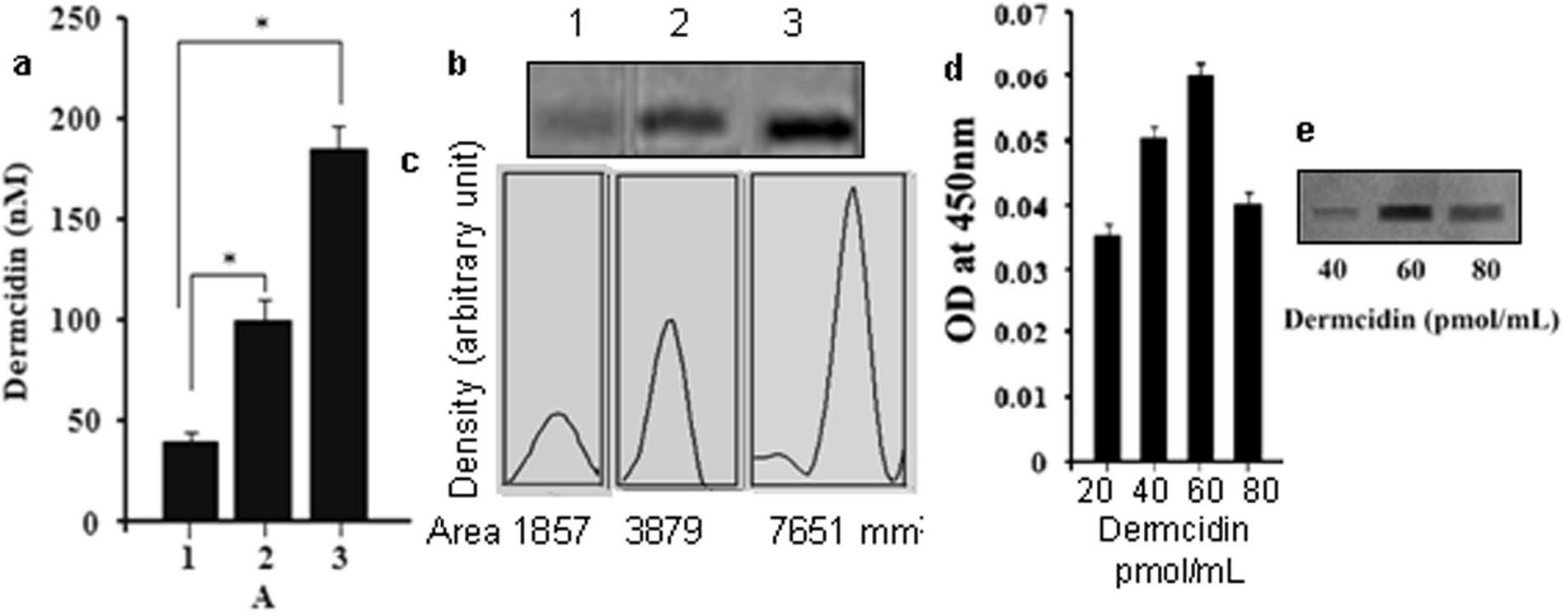
Identification of dermcidin in the cell free plasma of both normal and type-1 diabetes mellitus patients. The cell free plasma from both normal and diabetic patients was prepared as described in the Materials and Methods section. The amount of dermcidin was determined by ELISA by using polyclonal antibody raised against the purified dermcidin. Panel-a: Solid bar ‘1’ represents the dermcidin level in normal subject. Solid bar ‘2’ and ‘3’ represents the dermcidin level in T1DM patients with blood glucose level in between 120–340 mg/dL and > 340 mg/dL respectively. Panel-b: Immunoblot analysis of the plasma level of dermcidin in normal and T1DM subjects. ‘1’ represents the dermcidin band in the cell free plasma (CFP) of normal subject. ‘2’ and ‘3’ represents the dermcidin bands in the CFP of T1DM subjects with blood glucose level in between 120–340 mg/dL and >340 mg/dL respectively. Data are mean ± S.D. of at least 10 different experiments using 10 different samples from 10 different volunteers (Male = 5, Female = 5) (*represents p < 0.05). Panel-c: Integrated area of each of the immunopositive band as shown in the Panel-b. Panel-d: A standard bar diagram of ELISA assay for pure dermcidin protein. Dermcidin was purified by repeated gel electrophoresis as described. The ELISA assay of the purified dermcidin was conducted by using commercial dermcidin antibody. Panel-e: Western blot analysis of purified dermcidin by using commercial dermcidin antibody.

It was found that the patients with blood sugar level > 340 mg/dl had higher level of the stress induced protein (180 pmol/mL). Those T1DM patients who have blood sugar level in between 120–340 mg/dL the plasma DCN-2 level was 100 pmol/mL. But in the normal controls it was found that the plasma DCN-2 level was much less as compared to the T1DM subject (40 pmol/mL) (Fig. 1a).

It was found that the expression of DCN-2 immunopositive band was very high in the T1DM patients (Fig. 1c, lane-2 and 3). But in case of normal controls the expression of DCN-2 was less compared to the T1DM subject (Fig. 1b, lane- 1).

### Correlation between plasma dermcidin level with plasma NO and insulin levels in hyperglycemic and normoglycemic subjects

**Figure 2:**
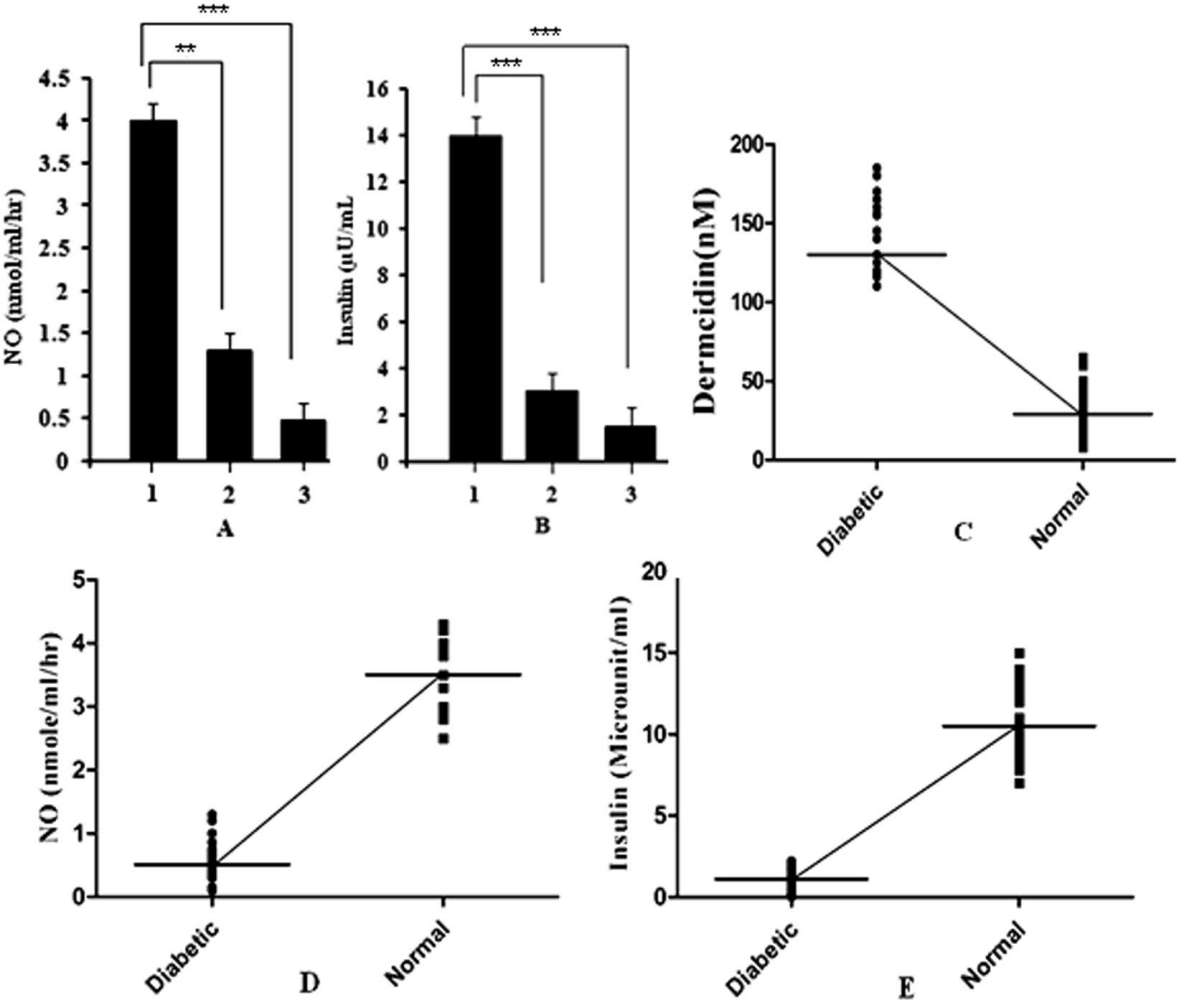
Plasma level of NO and insulin in normal and T1DM volunteers and their correlation with plasma dermcidin level: NO and insulin levels were measured by methemoglobin method and by ELISA in each volunteers as described in Materials and Methods. Panel-A: ‘1’ represents the plasma NO level in normal volunteers. ‘2’ and ‘3’ represents the plasma NO level in T1DM volunteers. Panel-B: ‘1’ represents the plasma insulin level in normal volunteers. ‘2’ and ‘3’ represents the plasma insulin level in T1DM volunteers. Panel-C, D and E represents highly and negatively correlation between plasma dermcidin and plasma NO and insulin level in T1DM and normal subjects. Each point represents the amount of insulin (µU/mL), NO production (nmol/mL/hr) and dermcidin level (nM) of at least 30 age and sex matched normal volunteers with T1DM volunteers (n = 30, male = 15, female = 15). (***Represents p < 0.0001, **represents p < 0.001).

It was noticed that T1DM subjects had lower levels of plasma NO and insulin when compared with the age and sex matched normal controls (Fig. 2A,B). It was also found that the plasma DCN-2 (Fig. 1a) and the plasma NO and insulin levels in T1DM were highly and negatively correlated (Fig. 2C,D and E), (coefficient of correlation ‘r’ = −0.9899). From Fig. 2D, it was found that plasma NO levels in diabetic patients (n = 30) were 0.5 nmol/mL (median ranging from 0.1 nmol/mL to 1.3 nmol/mL) and the plasma NO level in normal subjects were 4 nmol/mL (median ranging from 2.5 nmol/mL to 43 nmol/mL). Plasma insulin level in T1DM patients was 1.5 µU of insulin/mL (median ranging from 0.02 µU of insulin/mL to 2.2 µU of insulin/mL) and plasma insulin level in normal subject was 14 µU of insulin/mL (median ranging from 7 µU of insulin/mL to 13.7 µU of insulin/mL) (Fig. 2E).

### Plasma level of C-reactive protein (CRP) in both T1DM and normal subject

**Figure 3:**
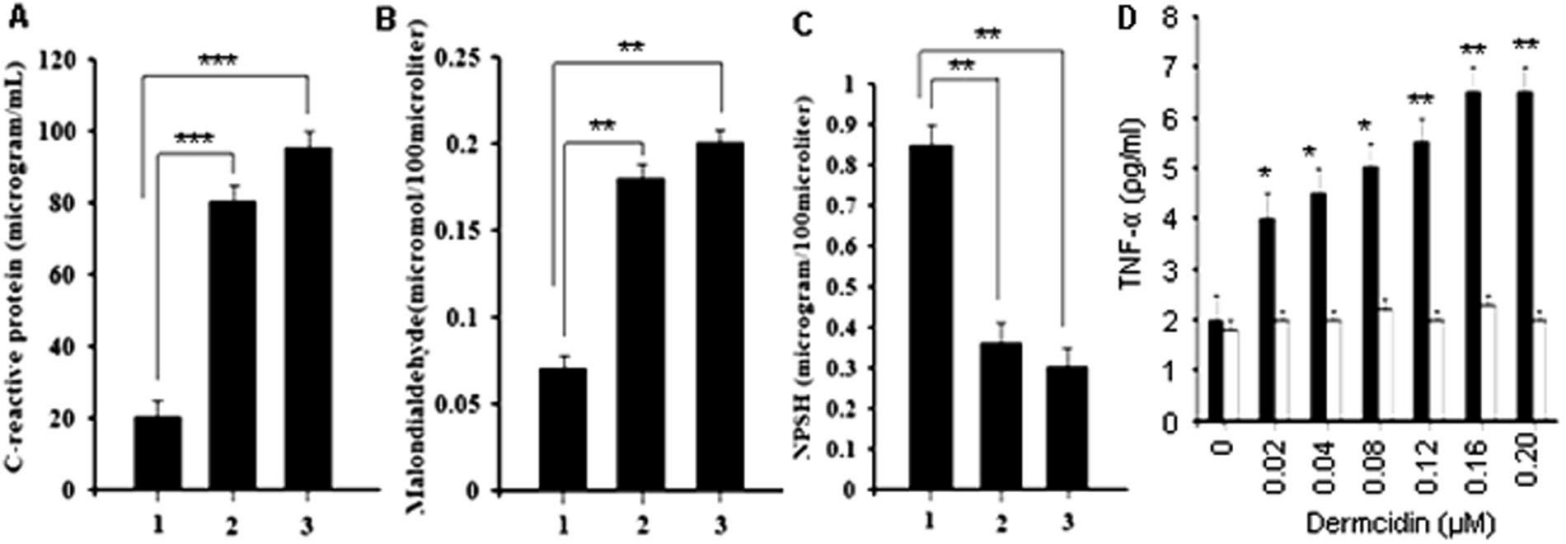
Plasma level of C-reactive protein, malondialdehyde and non-protein soluble thiol in T1DM and normal subjects and DCN-2 induced synthesis of TNF-α in liver cells: The amount of C-reactive protein, malondialdehyde and non-protein soluble thiol present in the CFP of T1DM and normal patients was determined as described in the Materials and Methods. Panel-A: Solid bar ‘1’ represents the amount of C-reactive protein present in the normal volunteers. ‘2’ and ‘3’ represents the amount of C-reactive protein present in the cell free plasma of T1DM volunteers Panel-B: Solid bar ‘1’ represents the amount of MDA present in the normal volunteers. ‘2’ and ‘3’ represents the amount of MDA present in the cell free plasma of T1DM subjects. Panel-C: Solid bar ‘1’ represents the amount of NPSH present in the CFP of normal volunteers. ‘2’ and ‘3’ represents the amount of NPSH present in the cell free plasma of T1DM subjects Panel-D: Solid bars (■) = DCN-2 induced synthesis of TNF-α in liver cell homogenate, hollow bars (□) = 0.9% NaCl induced synthesis of TNF-α in liver cell homogenate. (*Represents p < 0.05, ** represents p < 0.001, ***represents p < 0.0001).

It has been reported that the insulin sensitivity is negatively correlated with circulating high sensitive CRP (hs-CRP)^42^, and chronic inflammation due to activation of hs-CRP may play a role in changes in glucose homeostasis in T2DM^43^. It was found that patients with blood glucose level > 340 mg/dL had higher level of CRP in the plasma 90 µg/mL. Those patients who had blood sugar level in between 120–340 mg/dL, the plasma CRP level were 80 µg/mL. But in normal controls it was found that the plasma CRP level was much less 20 µg/mL compared to the T1DM subject (Fig. 3A).

### Plasma level of malondialdehyde (MDA) in both T1DM and normal subject

It was previously reported that inflammatory responses such as IL-6 and TNF-α accumulated oxidative damage products in the liver of diabetes mellitus (DM) patients^44^. It was found that plasma MDA level was 0.2 µmole/100 µL in the T1DM subject with blood glucose level > 340 mg/dL, which was very high than other groups (Fig. 3B).

### Plasma level of non-protein soluble thiol (NPSH) in both normal and T1DM subject

NPSH improve insulin resistance and exhibit significant reductions in serum free fatty acids, oxidative stress and inflammatory parameters in diabetic patients^45^. It was observed that normal patients had higher level of NPSH 0.85 µg/100 µL in their plasma where as diabetic patients have very low amount of NPSH 0.4 µg/100 µL in their plasma compared to the normal control (Fig. 3C).

### Effect of dermcidin isoform-2 in the synthesis of TNF-α in liver cell

Incubation of different concentrations of DCN-2 to the liver cell has shown to elevate TNF-α in the incubation mixture. It was found that the TNF-α level was increased from 2.59 ± 1.53 pg/mL to 6.8 ± 1.6 pg/mL due to incubation of 0.2 µM dermcidin to the liver cell homogenate for 60 min at 37 °C (Fig. 3D). The control had a very low TNF-α.

### Effect of injection of DCN-2 on blood glucose, NO and insulin level in normal adult mice

**Figure 4:**
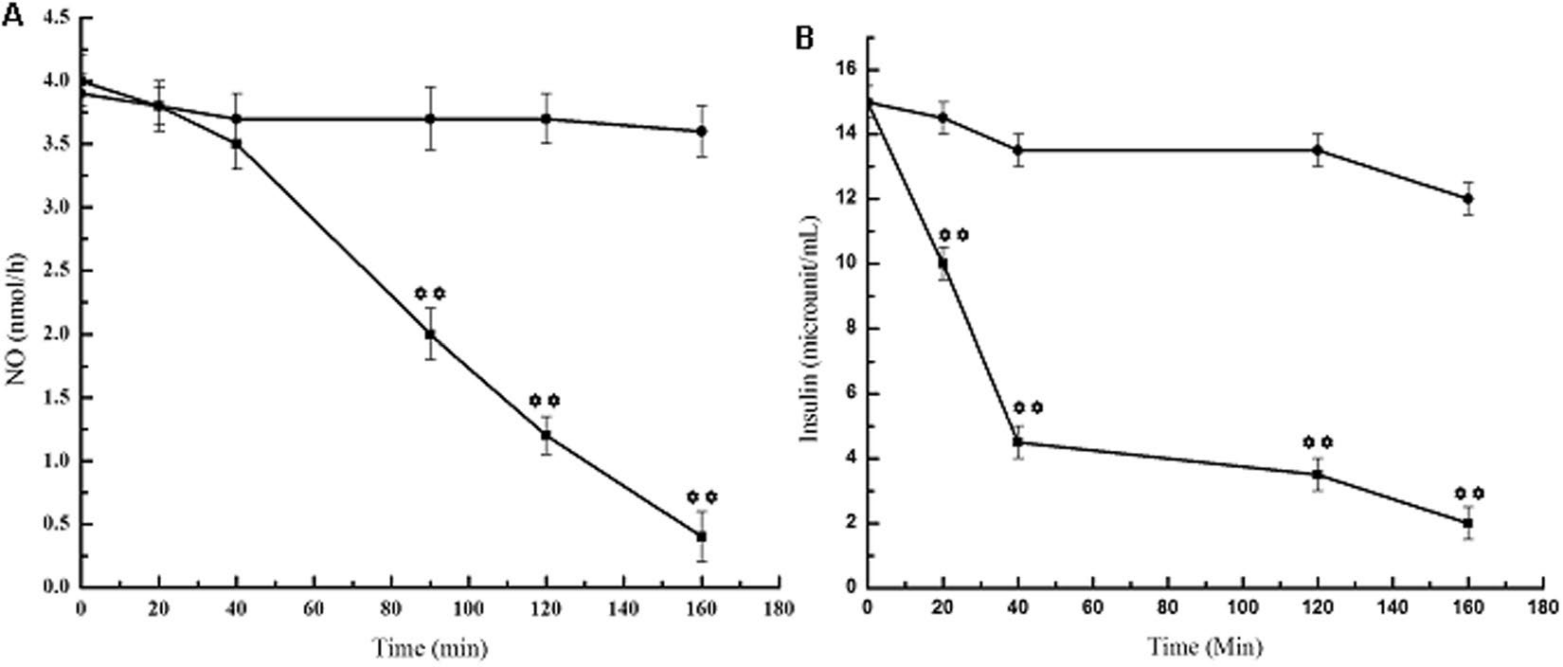
The *in vivo* effect of dermcidin on the blood glucose, NO and insulin level in normal adult mice: Purified DCN-2 was injected through tail vein into the circulation of normal mice, in the amounts similar to the plasma DCN-2 level in T1DM patients. The plasma glucose was determined by glucometer and plasma NO and insulin levels were determined by methemoglobin method and by ELISA as described in Materials and Methods at different time interval after the administration of DCN-2. Panel-A: Solid squares (■) = plasma NO level of normal mice after the treatment of DCN-2, solid circles (●) = NO level in normal mice after the treatment of 0.9% NaCl. Panel-B: Solid squares (■) = plasma insulin level in normal mice after treated with DCN-2 and solid circles (●) = plasma insulin level in normal mice after the treatment of 0.9% saline. The results are mean ± S.D. of at least 10 different experiments using 10 different diabetic mice (** represents p < 0.001).

It was found that the plasma glucose level of mice was increased from 100 ± 10 mg/dL (before DCN-2 was administered) to 160 ± 8 mg/dL at 90 min and to 350 mg/dL after 160 min (figure not shown). It was also found that, in control experiment, when the normal mice treated with 0.9% saline instead of DCN-2, the blood glucose level increased much less effectively (100 ± 10 mg/dL to 105 ± 8 mg/dL). When the plasma NO levels were determined in the same animal at different time interval, it was found that initial plasma NO level of 4.0 nmol/h in normal mice decreased to 2.0 nmol/h at 90 min and to 0.5 nmol/h after 160 min (Fig. 4A). But in control experiment where normal mice injected with saline the NO level decreased much less in plasma.

It was found that the plasma insulin concentration in the normal mice which was 15 ± 1.8 µU of insulin/mL before the administration of DCN-2 decreased to 4 µU of insulin/mL at 120 min and to 2.5 µU of insulin/mL after 160 min (Fig. 4B). But in control experiment where normal mice treated with saline the plasma insulin level was similar (15 ± 1.8 µU of insulin/mL) before and after the saline administration.

### Role of dermcidin on the inhibition of glucose activated nitric oxide synthase (GANOS)

**Figure 5:**
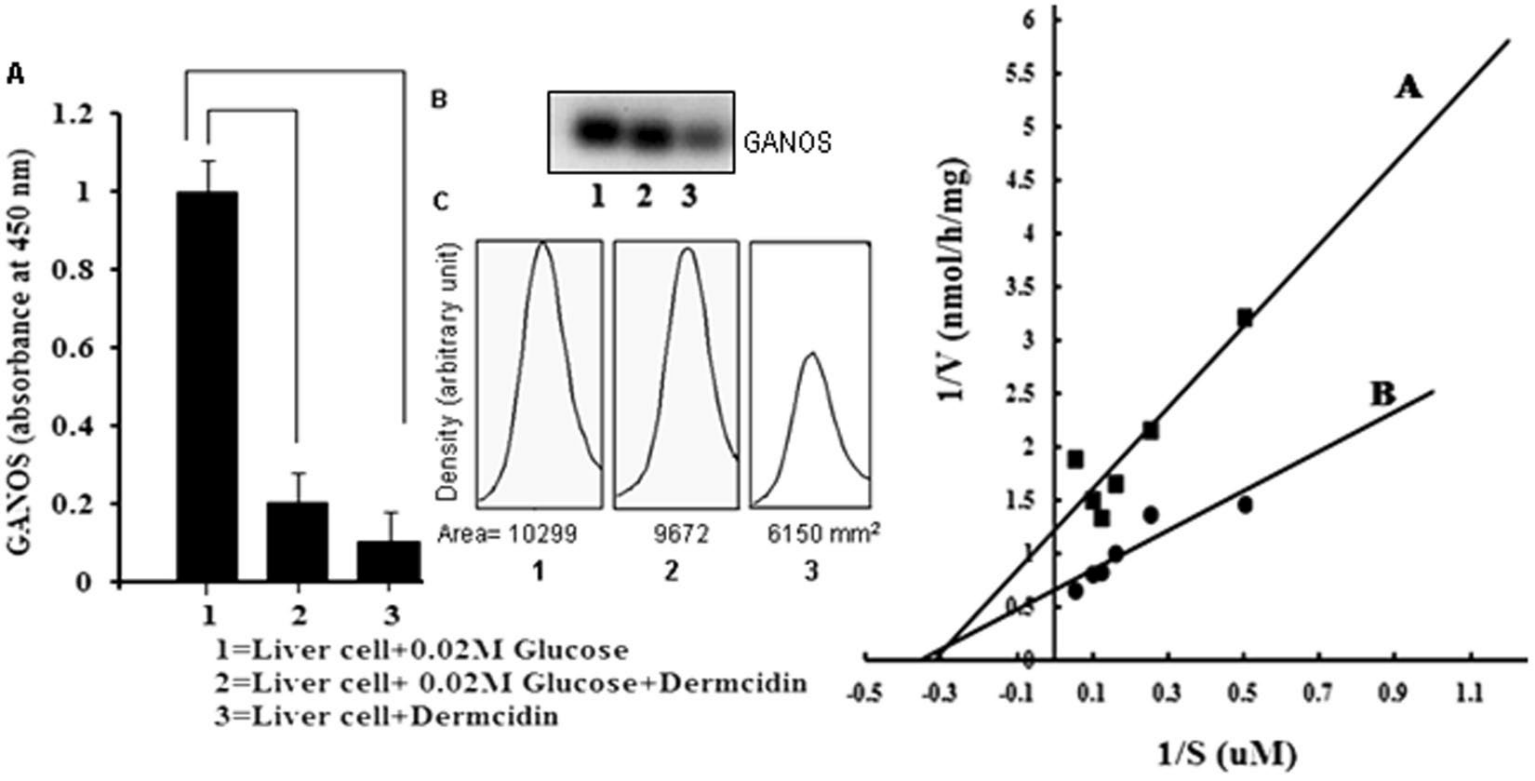
Decrease synthesis of glucose activated nitric oxide synthase (GANOS) by dermcidin: The liver cell homogenate was prepared in Tyrod’s buffer (pH 7.4) as described in the Materials and Methods. The liver cell homogenate was incubated with 0.02 M glucose in the presence and absence of DCN-2 for 30 min at 37 °C. In each case GANOS synthesis was determined by ELISA by using GANOS antibody as described. Panel-A: ‘1’ represents GANOS synthesis in liver cell homogenate in the presence of 0.02 M glucose. ‘2’ represents GANOS synthesis in liver cell homogenate in presence of both glucose and DCN-2 and ‘3’ represents synthesis of GANOS in liver cell homogenate in presence of only DCN-2. Panel-B: Immunoblot analysis of the GANOS in liver cell homogenate. ‘1’ represents the expression of GANOS immunopositive band in liver cell homogenate incubated with glucose only, ‘2’ represents the expression of GANOS band in liver cell homogenate in the presence of both glucose and DCN-2 respectively and ‘3’ represents the expression of GANOS immunopositive band in liver cell homogenate in the presence of DCN-2 only. Data are mean ± S.D. of at least 5 different experiments using 5 different animals each in triplicate (* represents p < 0.05). Panel-C: Integrated area of each of the immunopositive band as shown in the Panel-B. Panel-D: Lineweaver Burk plot of the inhibition of nitric oxide synthase activated by glucose in the liver cell membrane of adult mice. Mice liver membrane was prepared in Tyrod’s buffer (pH 7.4) as described in the Materials and Methods section. Lineweaver-Burk plot was constructed by adding different amounts of l-arginine to the reaction mixture containing 0.02 M glucose in the presence and absence of 0.2 µM DCN-2. Line A represents the formation of NO in the presence of glucose, and the line B represents the formation of NO in the presence of both glucose and dermcidin. Each point represents mean of 5 different experiments each in triplicate.

In the present study it was explored that addition of DCN-2 in the hepatocyte suspension, in the amounts similar to the plasma DCN-2 level in the T1DM victims had blood glucose level > 340 mg/dL, totally obliterated the synthesis of GANOS even if the presence of 0.02 M glucose (Fig. 5A).

It was found that addition of DCN-2 to the hepatocyte suspension inhibited the expression of cNOS immunopositive band (Fig. 5B,C, lane-3). Addition of 0.02 M glucose along with DCN-2 slightly increased the expression of cNOS immunopositive band and the integrate area of the band was 9672 mm^2^ (Fig. 5C, lane-2), which was maximally stimulated by the addition of 0.02 M glucose only to the liver cell homogenate (Fig. 5B, lane-1).

The DCN-2 induced inhibition of cNOS leading to the inhibition of NO production from *l-arginine* was further determined by adding dermcidin to the liver cell homogenate treated with 0.02 M glucose in the presence of *l-arginine*. Lineweaver-Burk plot of the GANOS in the supernatant from the liver cell homogenate in the presence of glucose that resulted in the stimulation of NO synthesis in the reaction mixture as described in Fig. 5D demonstrated that the Km of GANOS was 3.33 µM with the maximum velocity (Vmax) of 2 nmol/mg protein/h (line-B). The addition of 0.2 µM DCN-2 to the reaction mixture increased the Km from 3.33 µM to 4.16 µM arginine with concomitant decrease of the Vmax from 2 nmol/mg protein/h to 0.83 nmol/mg protein/h (line-A) indicating that the rate of synthesis of NO in the presence of glucose was decreased by nearly 2.5 times in the presence of 0.2 µM DCN-2 *in vitro* (Fig. 5D).

### Effect of dermcidin on glucose uptake, insulin release and Glut-4 expression

**Figure 6:**
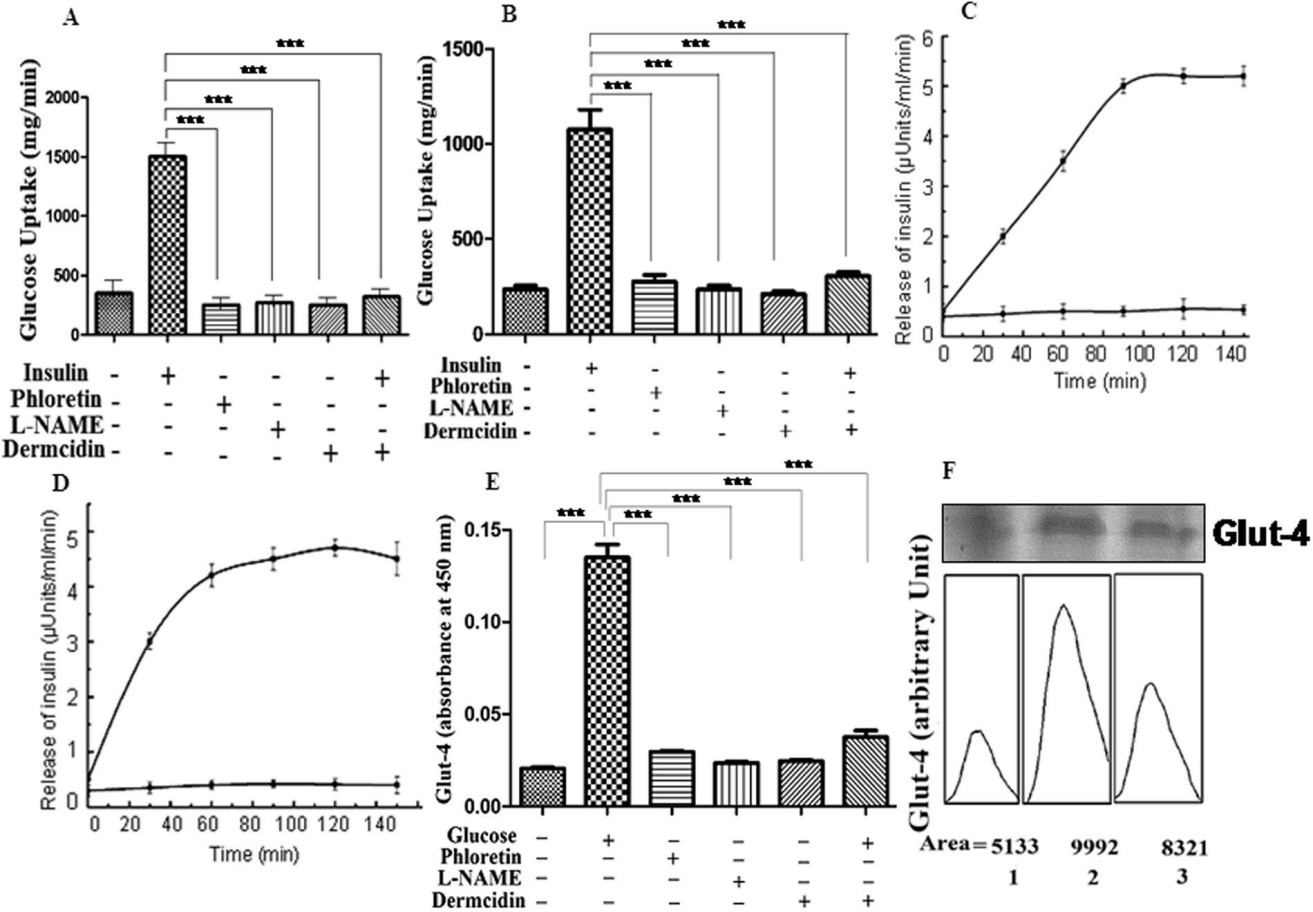
Role of dermcidin in glucose uptake, insulin release and glut-4 expression: Panel-A: The uptake of 2-deoxy-D ^14^C glucose by insulin was measured in liver cell pretreated with either dermcidin, phloretin or L-NAME. Panel-B: The uptake of 2-deoxy-D 14 C glucose by insulin was measured in pancreatic β-cell pretreated with dermcidin, phloretin or L-NAME. Panel-C: Release of insulin from liver cells in the presence and absence of dermcidin. Solid squares (■) = insulin release in the absence of DCN-2, solid circles (●) = insulin release in the presence of DCN-2. Panel-D: Release of insulin from pancreatic β-cells in the presence and absence of dermcidin. Solid squares (■) = insulin release in the absence of DCN-2, solid circles (●) = insulin release in the presence of DCN-2. Panel-E: Glut-4 synthesis in liver cells in presence of glucose, dermcidin, phloretin or L-NAME. Panel-F: Immunoblot of Glut-4 in liver cells treated with dermcidin and in the presence and absence of glucose and the integrated area of each of the Glut-4 immunopositive band. Data are mean ± S.D. of at least 5 different experiments using 5 different animals each in triplicate (*** represents p < 0.0001).

It has been reported that insulin-induced glucose uptake was facilitated through NO, a second messenger molecule of insulin^46^. Experiments were carried out to determine the role of DCN-2 on glucose uptake. It was found that addition of 0.2 µM DCN-2 completely inhibited the glucose uptake in liver and pancreatic β-cells (Fig. 6A and B). DCN-2 also inhibited the release of insulin from both these cells into the circulation to maintain the normal glucose homeostasis (Fig. 6C and D). It was found that the reaction mixture containing pancreatic islets of Langerhans when incubated with 0.02 M glucose stimulate insulin synthesis from the basal 0.008 ± 0.06 µU of insulin/mL to 0.15 ± 0.06 µU of insulin/mL. Addition of 0.2 µM DCN-2 in the reaction mixture resulted 60% inhibition of insulin synthesis (0.06 µU insulin/mL). As evident from Fig. 6E and F, DCN-2 induced inhibition of glucose uptake was mediated through the DCN-2 induced inhibition of Glut-4 synthesis in liver cells. Glut-4 synthesis was maximally stimulated by 0.02 M glucose.

### Dermcidin inhibit GLUT-4 and insulin gene expression in hepatocytes

**Figure 7:**
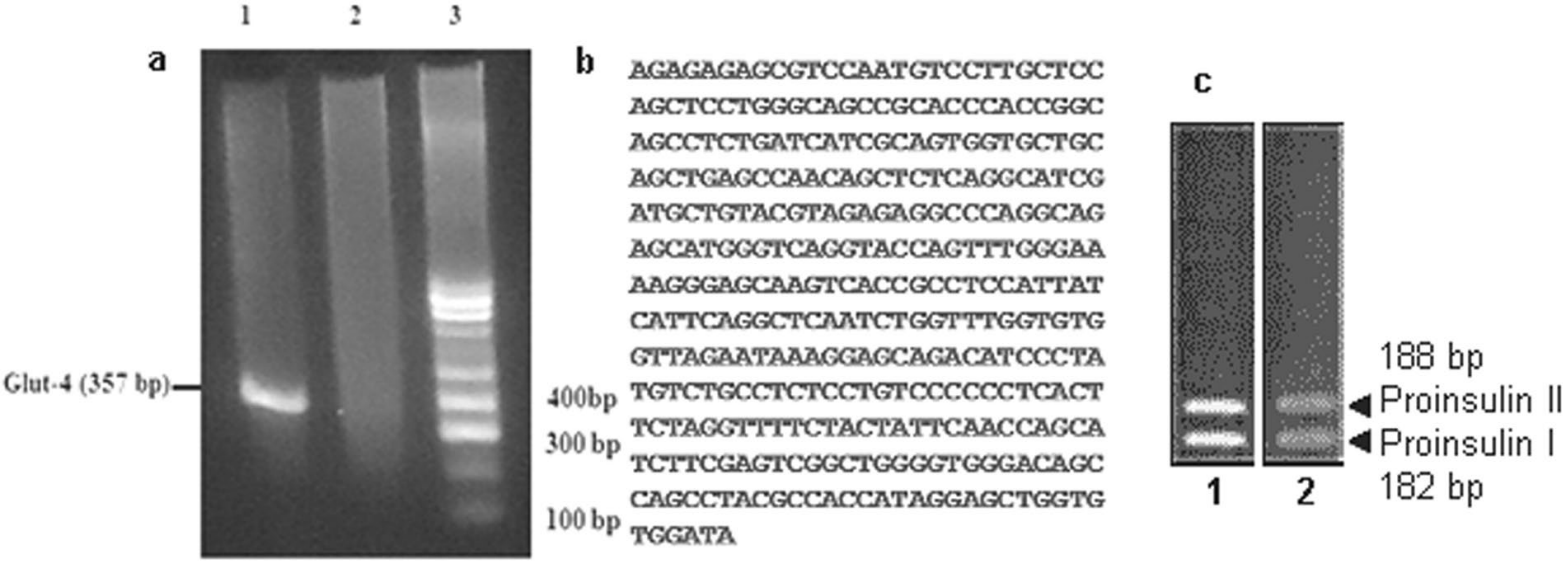
Role of dermcidin in Glut-4 and insulin gene expression. The liver cell homogenate was incubated with 0.02 M glucose in the presence and absence of 0.2 µM DCN-2 for 30 min at 37 °C. cDNA was prepared from the liver cells by RT-PCR method. Panel-a: Agarose gel electrophoresis for Glut -4 gene expression. Lane-1 represents the Glut-4 gene (357 bp) in the liver hepatocytes in presence of glucose; Lane-2 represents the no expression of Glut-4 gene in the liver hepatocytes in presence of both glucose and DCN-2 and Lane-3 represents the standard base-pair marker. Panel-b: The sequence of cDNA from the liver hepatocytes of mice incubated with glucose representing Glut-4 gene. The sequence was matched with the known DNA sequence of mice Glut-4 gene and the alignment score was >85%. Panel-c: Agarose gel electrophoresis of pro-insulin genes (cDNAs) in liver cells. Lane-1 represents the expression of pro-insulin genes I and II in the presence of 0.02 M glucose. Lane-2 represents the reduced expression of pro-insulin genes in the presence of both glucose and DCN-2. Results shown are representatives of the optical densities obtained from 6 different experiments using 6 different animals.

The results described in Fig. 6E and F demonstrate that DCN-2 induced inhibition of glucose uptake in liver cell was mediated through the inhibition of Glut-4 synthesis. It is necessary to determine the role of DCN-2 on gene level. It was found that glucose induced NO production through the activation of cNOS (GANOS) in hepatocytes was not only involved in the increased synthesis of GLUT-4 in liver cells, but it also resulted in the increased expression of GLUT-4 gene in the hepatocytes (Fig. 7a, lane-1) by RT-PCR method. But when the liver cell homogenate was incubated with both glucose and 0.2 µM DCN-2, the expression of GLUT-4 gene was completely inhibited (Fig. 7a, lane-2). The nucleotide sequence of Glut-4 gene was determined (Fig. 7b) and alignment of the sequence was matched with that of the known DNA sequence of mice Glut-4 gene. It was found that, in the mice hepatocytes the Glut-4 gene alignment score was >85%. These results suggested that glucose induced synthesis of Glut-4 in the hepatocytes was probably mediated through the Glut-4 gene up regulation.

It was also found that incubation of liver cell homogenate with glucose showed the expression of both pro-insulin genes I and II as determined by cDNA analysis (Fig. 7c, lane-1), which was expressed due to NO synthesis by the glucose induced activation of cNOS (GANOS). But when the liver cell homogenate was incubated with both glucose and 0.2 µM DCN-2, the expression of both pro-insulin genes I and II was reduced (Fig. 7c, lane-2).

## Discussion

These results suggested that environmental stresses, oxidative stresses, reduced production of anti-oxidants, products generated from lipid peroxidation and autoimmunity are key factors in the genesis of both type-1-diabetes mellitus (T1DM) and type-2-diabetes mellitus (T2DM). It has been previously reported that autoimmunity itself has an important role in the destruction of the pancreatic β-cells leading to T1DM^8, 47^, but the role of autoimmunity in the stress induced pathogenesis of T1DM was less understood. We have recently reported that a stress induced protein identified to be dermcidin isoform-2 (DCN-2) appeared in the circulation of T1BDM patients which lack immunological markers^10^. It supports our present finding (Fig. 1) and it is also reported that DCN-2 inhibit insulin synthesis not only in pancreatic β-cells^32^, but also in the hepatic cells of adult mice^32^. In this context, it should be mentioned here that our result as described in Fig. 2 demonstrated a negative correlation between plasma DCN-2 level and plasma insulin and NO level in diabetes patients and a positive correlation between DCN-2 level and blood glucose level in those patients.

DCN-2 induced inhibition of insulin synthesis in the pancreatic β-cells was mediated via the impairment of glucose uptake in these cells^11^. It has been reported that for the synthesis of insulin in the liver cells, activation of a constitutive nitric oxide synthase (cNOS) by glucose was required^33^. The synthesis of NO in the liver cells via the activation of cNOS by glucose, (GANOS) directs the synthesis and translocation of Glut-4 to the liver membrane peripheries for the transportation of the sugar into the hepatic cells. This will lead to the production of insulin through the expression of pro-insulin genes^4, 33^. We report herein that liver cells treated with DCN-2 at a level, similar to the plasma DCN-2 level of T1DM patients having blood glucose level > 340 mg/dL completely obliterated the GANOS synthesis in liver cells (Fig. 5A). It was also found that treatment of liver cell homogenate with DCN-2 reduced the expression of immuno-positive bands of GANOS (Fig. 5B). In a separate experiment, it was also found that the DCN-2 induced decrease of the plasma NO level in normal mice (Fig. 4A) was a consequence of the inhibition of a constitutive NOS (cNOS) activated by glucose in the liver cells of mice (Fig. 5D). Lineweaver Burk plot of the DCN-2 induced effect on glucose activated cNOS inferred that dermcidin act as a competitive inhibitor to the enzyme and was found to compete with *l-arginine*, the substrate of NOS (Fig. 5D). Similar results were also obtained using 0.2 mM phloretin in the inhibition of glucose activated cNOS experimentation (unpublished). In this sense, DCN-2 was found to be effective in the inhibition of insulin synthesis (Fig. 4B) through the inhibition of GANOS in the liver cells (Fig. 5). Our result also suggests the relationship between increased levels of DCN-2 and decreased levels of NO production that ultimately led to the development of insulin resistance (IR) or type-2 diabetes mellitus (T2DM). Jiang *et al*. showed that T2DM is associated with impaired NO production and reduced Glut-4 translocation^48^. Impaired NO production, a phenotype of IR has also been associated with cardiovascular disease (CVD)^49, 50^. It was also reported that NO has a role in glucose transport and metabolism in rat skeletal muscle through NOS activation^51^ and the development of T2DM is preceded by the defects in both insulin-dependent and insulin-independent glucose uptake^52^. Our results demonstrated that pre-incubation of liver and pancreatic β-cells with 0.2 µM DCN-2 completely inhibited the insulin induced uptake of glucose in these cells (Fig. 6A,B), which further inhibited the release of insulin from both the liver and pancreas (Fig. 6C,D). The additive effect generated from this vicious circle make the diabetic condition more severe. In liver cells, DCN-2 induced inhibition of glucose uptake was mediated by the DCN-2 induced inhibition of Glut-4 synthesis (Fig. 6E), due to the down-regulation of Glut-4 gene expression (Fig. 7a). However, the mechanism of DCN-2 induced inhibition of glucose uptake in pancreatic β-cells is still obscure.

The current results also demonstrated that DCN-2 act as an inhibitor of glucose-induced NOS (Fig. 5D). In the current study, DCN-2 impaired the synthesis of insulin protein and its action (Fig. 4B) due to the impairment in the expressions of pro-insulin genes I and II (Fig. 7c), and in the production of NO (Fig. 4A). This suggests that dermcidin plays a crucial role in IR through the inhibition of GANOS which further inhibit the glucose uptake via decreased synthesis of Glut-4.

Abnormal nutrient metabolism is one of the consequences of diabetes. Atherosclerosis was reported to be the major cause of increased occurrence of cardiovascular and cerebrovascular disorders. Again, hypertension and diabetes mellitus both type-1 and type-2 are reported to be the major risk factors for the genesis of atherosclerosis^21^, The development of T1DM due to both increased DCN-2 level and DCN-2 induced inhibition of NO and insulin synthesis might promote prothrombotic condition that leads to atherosclerosis. In this context, it should be mentioned that DCN-2 was found to aggregate platelet at nM concentration through the inhibition of both NO and insulin synthesis^11^. As the level of DCN-2 was very high in T1DM individuals (Fig. 1), it could predict that DCN-2 induced impairment of systemic NO- synthesis and insulin-synthesis (Fig. 4A,B) might play an essential role in the development of atherosclerosis in T1DM patients. Indeed, no decisive mechanism related to the generation of atherosclerosis is currently available.

The human dermcidin isoform-2 was reported to be a potent inhibitor of all known forms of nitric oxide synthase (NOS)^31^ because it acts as a competitive inhibitor of l-arginine the substrate of NOS. The protein DCN-2 has 6 Arg in its amino acid sequence (GenBank: ABQ53651.1). This has also been demonstrated from the laboratory of our research group^11^. The Swiss-model 3-D structure of this protein suggests that these Arg are distributed at regular interval and the 3 Arg are present in the central position (53, 59 and 62) of this protein (figure not shown). This Arg may have some role in blocking the substrate binding site of NOS. In this respect, it should be important to mention that impairment of insulin functions or insulin resistance may lead to hypertension, but no mechanistic information is yet available on the development of this disease. We have reported that DCN-2 was not only involved in the development of atherosclerosis in T1DM as a diabetogenic agent, but the protein was also involved in the genesis of primary or essential hypertension through the impairment of renal r-cortexin synthesis in kidney cortex cells due to the inhibition of systemic NO synthesis. So, our results might suggest that DCN-2 induced inhibition of systemic NO synthesis both in animal model (Fig. 4A) and T1DM individuals (Fig. 2A) could be involved in the development of essential hypertension.

**Figure 8:**
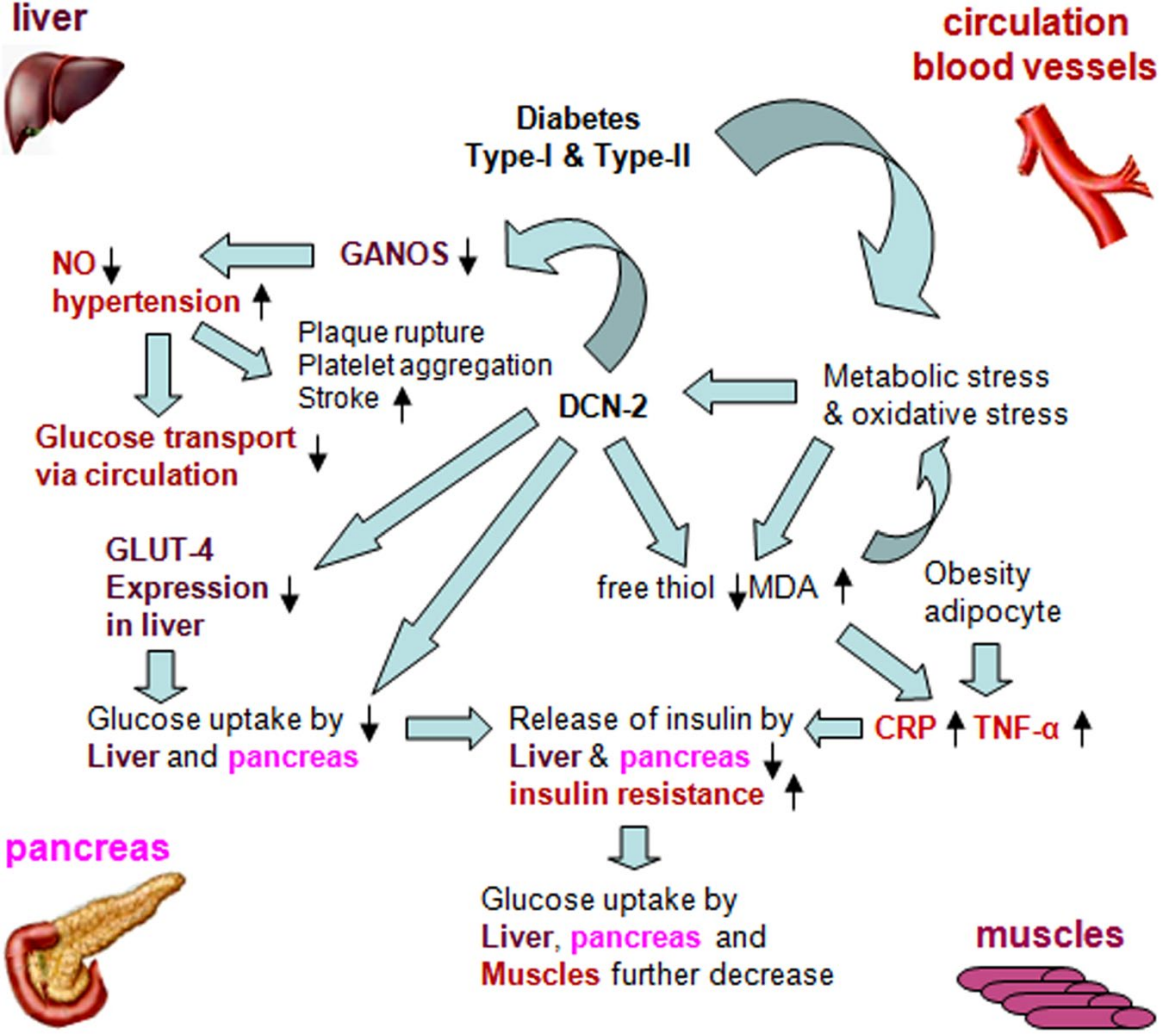
Diagrammatic representations of the role of human dermcidin isoform-2 (DCN-2) in the initiation of different metabolic dysregulations. Stress-induce protein DCN-2 disrupts glucose and lipid homeostasis by down-regulating NOS expression and up-regulating CRP and TNF-α expression.

A recent report suggested that an effective innate immune response can inflate obesity-induced inflammation and various metabolic disorders including diabetes^53^. Inflammatory markers such as TNF-α, IL-6 and adipocyte metabolism play an essential role in changes in glucose homeostasis in T2DM individuals^44^. Recent studies revealed that TNF-α may be a very important molecule that is produced by fat cells in obesity and interferes with insulin action^54, 55^. Infusion of TNF-α to normal rat led to the development of severe hepatic and peripheral insulin resistance^56^. We have found that most of the diabetic patients show higher level of TNF-α in their plasma and incubation of liver cells with 0.2 µM DCN-2 shows significant elevation of TNF-α synthesis through the up-regulation of TNF-α mRNA by DCN-2 (Fig. 3D). In this respect, it should be mentioned that TNF-α inhibits insulin stimulated glucose transport via the down-regulation of Glut-4 protein^57^ which is evident in our current investigation (Fig. 6). This is also supported by the results in the present studies (Fig. 3D). That may result in reduced secretion of insulin from liver and ultimately develop IR and T2DM. Balance between hepatic glucose uptake (HGU) and hepatic glucose production (HGP) has an important role in the regulation of glucose homeostasis, in particular in the post absorptive state. We have found that severity of hyperglycemia is associated with increased high-sensitive C-reactive protein (hs-CRP) in the cell free plasma of diabetic individuals (Fig. 3A). C-reactive protein is regarded as a metabolic inflammatory marker. Taking into the account the note on the TNF-α and IL-6 increase, it may be hypothesized the stress-induced metabolic dysregulations might initiate inflammatory responses. Moreover these two have some interactive relation in the fastening the diabetic pathogenesis. The brief diagrammatic representation is made in the Fig. 8. This result suggest that a significant association between metabolic syndrome component with hs-CRP. It was also reported that depletion of serum total biliruvin (TB) is associated with the enhancement of inflammatory responses and oxidative stresses in T2DM individuals^58^ which put severe strain on pancreatic β-cells^53^. The reason of pancreatic β-cells become less responsive is due to the down-regulation of glucose-sensing mechanism in the β-cells (glucotoxicity) leading to diminished insulin secretion. Lower TB may also be the result of impaired liver function^58^. A certain degree of impaired liver function may develop insulin resistance. We have also found that most of the diabetic patients showed lower level of plasma non-protein soluble thiol (NPSH) with higher level of oxidative stress marker malondialdehyde (MDA) (Fig. 3B,C) and lipid components (unpublished). Higher level of MDA indicates lipid peroxidation in the subjects with both T1DM and T2DM^59^. High MDA level is responsible for chronic hyperglycemia either due to lower insulin secretion or due to insulin resistance through the generation of excess free radicals^60^. Our results also demonstrated a positive correlation between high plasma MDA and CRP level with high DCN-2 level in T1DM victims. These suggest that abnormalities in glucose metabolism may impair lipid metabolism, thereby linking obesity to non-insulin dependent diabetes mellitus (NIDDM) or T2DM. But the mechanism that obesity leads to insulin resistance and develop NIDDM or T2DM remains speculative.

In the current study mouse was used to test the human DCN effects. Homolog-protein exact similar to human DCN, is absent in mouse. It is reported that the DCN, an antimicrobial peptide that is secreted by sweat glands, is a human homolog of mouse proteolysis-inducing factor (PIF)^61^. The human DCN has been shown to increase TNF-α in obese mouse model with ~30% decrease in insulin level. A certain degree of insulin-resistance has also been demonstrated in human DCN-treated mouse model^62^. In our study, beside similar response in TNF-α and insulin level, we further demonstrated very important roles of DCN in glucose homeostasis by influencing GLUT-4, NOS, NO and other relevant factors in mouse tissue. Not only mouse, several other far-coated mammals don’t express exact homolog to human DCN. But, very much similar types of proteins (with similar kind of functions i.e. anti-microbial etc.) designated by different names are synthesized in these animals. It has been reported that the N-terminal 30 amino acid peptide of DCN, which is known as either survival promoting peptide, diffusible survival evasion peptide (DSEP), or Y-P30^63, 64^, promotes neural cell survival under oxidative conditions. Human DCN is also demonstrated as oxidative-stress induced protein. The different roles of the gene products of DCN suggest that DCN has antimicrobial and oncogenic functions, as well as a wide range of biological functions. In this context, it should be mentioned here that native DCN is rapidly degraded by proteolytic enzymes. These features are similar to natively intrinsically unstructured proteins. The precise nature of the post translational modifications of human DCN, have not been fully elucidated. DCN protein was composed of several different peptides. It is likely that differential proteolysis is responsible for the production of the different DCN peptides. However, the specific proteases involved in differential proteolysis remain obscure. These proteins exhibit different structural conformations (i.e. lesser globularity, compactness and secondary structure)^65^ and post translational modifications^66^, which is accounted for their important and diverse biological functions. In our current study, human DCN-induced increase in inflammatory markers like c-reactive protein and TNF-α has been demonstrated. This study and some of our previously reported study decisively demonstrate important roles of human DCN in nutrient/energy metabolism/ glucose homeostasis, in cardiovascular physiology and in tumerogenesis and carcinogenesis^10, 12, 31, 32^. The DCN-associated interactive metabolic machinery is unlikely to be available in mouse. Hence, this null condition (without the influence of host DCN) serves as a good mammalian environment to test human DCN in mouse model^62^. Further, taken into account the fact that mouse NOS, insulin and GLUT-4 are very good homologue to that of human corresponding proteins. It can be hypothesized that the effects noticed in mouse is due to the application of exogenous human-DCN.

The present findings on insulin and GLUT-4 gene expressions data may suggest that dermcidin might have some role on transcriptional regulations. Role of transcription factors and/or nuclear receptors might be involved in glucose and DCN mediated gene regulations. Roles of liver in glucose homeostasis are evident^67^. Gene regulations by glucose via transcription factor ChREBP and ligand-activated nuclear receptor LXR have been demonstrated^67^. Influences of hepatic gene expression and energy-metabolism by other nutrients may be controlled by the nuclear receptors PPARα and FXR^68^. The evaluation and the potential role of NR4A orphan nuclear receptor families have been demonstrated in the regulation of glucose homeostasis and the development of type 2 diabetes^69^. The involvement of the DCN on nuclear receptors actions has not been investigated. The protein Y-P30 having distinct N-terminal homology to human DCN has been shown to control the nuclear localization of Calcium/calmodulin-dependent serine kinase (CASK) with its binding partner syndecan (SDC)^70^. Dermcidin derived peptide has been shown to inhibit bacterial macromolecular synthesis, especially RNA and protein synthesis, without binding to microbial DNA or RNA^71^. The mechanism of DCN induced down regulations of eukaryotic genes need to be explored.

Oxidative stress is one of the main applicators for induction of human DCN. In normal physiological conditions a certain level of ROS is regularly produced in the system during normal physiological function or as a metabolic by-product during the cellular respiration mainly by the mitochondrial processes. Hydrogen peroxides and free radicals like superoxide anion etc. have some functions during normal physiological processes as well as in pathological/ infection conditions. Transition metals (i.e. Fe and Cu) which are abundant in the biological system are the potent reactant for H_2_O_2_ and free radicals to generate radical-cascade reactions resulting in a number of byproducts^72^. Report reveals that increased extracellular glucose (30 mmol/L) can rapidly stimulate the generation of intracellular ROS through NADPH oxidase and mitochondrial pathways^73^. High glucose exposure and cytokine-treatment enhanced the generation of ROS and activation of inflammatory and apoptotic responses in endothelial cells^74^. ROS in turn can generate the pathological conditions associated with diverse human inflammatory diseases. Stress-induced proteins like antimicrobial peptides/dermcidin have a role to sense the status of the redox balance^75^.

Taken together, the above results suggested that the effect of stress induced protein dermcidin and the chronic inflammatory products TNF-α, metabolic inflammatory molecules C-reactive protein and oxidative stress inducer malondialdehyde are involved in the pathogenesis of both T1DM and T2DM. This state ultimately led to the development of acute ischemic heart disease (AIHD), which in turn could be overcome by systemic increase of insulin as reported to be a multifaceted antithrombotic humoral factor^76^.

## Electronic supplementary material

PAGE picture of purified human dermcidin (DCN) protein
