# Supplementary material for: The role of Dermcidin isoform-2 in the occurrence and severity of Diabetes
Source: Sci Rep. 2017 Aug 15;7:8252. doi: 10.1038/s41598-017-07958-3 (PMC5557962; doi:10.1038/s41598-017-07958-3)
Supplement: Supplementary file 1 — PAGE picture of purified human dermcidin (DCN) protein [file 41598_2017_7958_MOESM1_ESM.pdf]

Title: The role of Dermcidin isoform-2 in the occurrence and severity of Diabetes

Authors: Suman Bhattacharya[1,2], Mobidullah Khan[2], Chandradipa Ghosh[3], Sarbashri Bank [1,2], Smarajit Maiti[2*]

1. Sinha Institute of Medical Science and Technology, West Bengal, India
2. PG Department of Biochemistry, Cell and Molecular Therapeutics Laboratory,

   Oriental Institute of Science and Technology, Midnapore, West Bengal, India.
3. Department of Human Physiology with Community Health, Vidyasagar University, Midnapore, West Bengal, India

***Corresponding Author**
Dr. Smarajit Maiti
Head, Department of Biochemistry and Biotechnology
Cell and Molecular Therapeutics laboratory, OIST, Vidyasagar University
West Bengal, Midnapore, India
Email: maitism@rediffmail.com
Phone- 9474504269

Supplementary figure

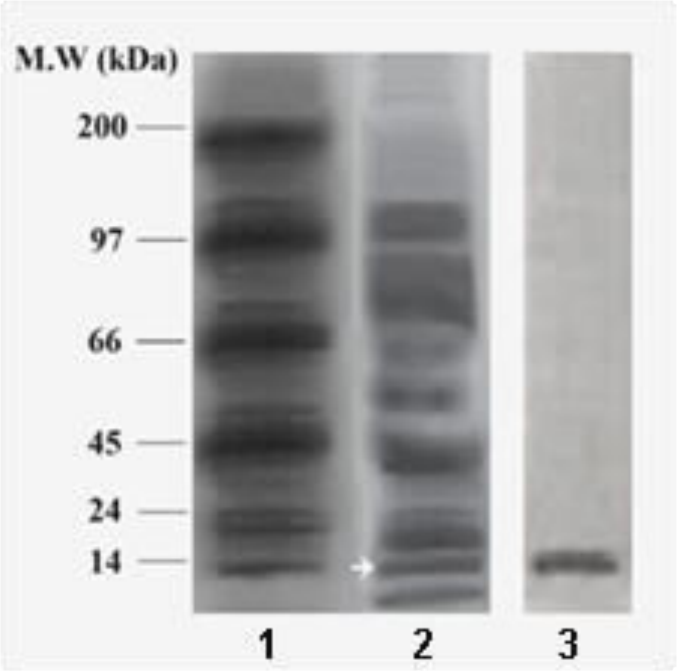

Representative picture.
Polyacrylamide gel electrophoresis of diabetic/AMI human plasma sample and purified DCN protein.
Lane 1: marker, 2: plasma sample of severe diabetic/AMI individual, triturated (from gel) protein band from DCN position which was used for further investigation.
